# Stress hyperglycemia ratio as a prognostic indicator for long-term adverse outcomes in heart failure with preserved ejection fraction

**DOI:** 10.1186/s12933-024-02157-7

**Published:** 2024-02-13

**Authors:** Abdul-Quddus Mohammed, Yongqiang Luo, Kaitao Wang, Yang Su, Lu Liu, Guoqing Yin, Wen Zhang, J Jiasuer. Alifu, Redhwan M. Mareai, Ayman A. Mohammed, Yawei Xu, Fuad A. Abdu, Wenliang Che

**Affiliations:** 1 Department of Cardiology, Shanghai Tenth People’s Hospital, Tongji University School of Medicine, 301 Yanchang Road, Shanghai, 200072 China; 2 Department of Cardiology, Shanghai Tenth People’s Hospital Chongming branch, Shanghai, China

**Keywords:** Heart failure with preserved ejection fraction, Stress hyperglycemia ratio, Clinical outcomes

## Abstract

**Background:**

Recent studies highlighted that stress hyperglycemia ratio (SHR) is a potential predictor for future risk in heart failure (HF) patients. However, its implications specifically in HF with preserved ejection fraction (HFpEF) are not yet fully elucidated. We aimed to investigate the association between SHR and long-term clinical outcomes in HFpEF patients.

**Methods:**

HFpEF patients enrolled between 2015 and 2023, were followed (mean 41 months) for a composite outcome of all-cause, cardiovascular mortality, and HF rehospitalization. SHR was established as the ratio of acute-chronic glycemia from admission blood glucose and glycated hemoglobin. The optimal cut-off for SHR to predict outcomes based on event prediction was determined through ROC analysis, and the cutoff was identified at 0.99. The effect of SHR on adverse risk was examined through the Cox hazards and Kaplan-Meier survival methods. A Pearson correlation analysis was conducted to assess the relationship between SHR and the severity of HF, as indicated by N-terminal pro-brain natriuretic peptide (NT-proBNP) levels. Furthermore, the incremental prognostic value of SHR was further assessed by the integrated discrimination improvement (IDI) and the net reclassification improvement (NRI).

**Results:**

Among the 400 enrolled patients, 190 individuals (47.5%) encountered composite events over the 41-month follow-up period. SHR was significantly elevated in patients with events compared with those without (*p* < 0.001). All patients were stratified into high SHR (*n* = 124) and low SHR (*n* = 276) groups based on the SHR cutoff. The high SHR group had a significantly higher incidence of adverse events than the low SHR group (log-rank; *p* < 0.001). Additional analysis indicated a poorer prognosis in patients with low left ventricular EF (LVEF) levels (50 < LVEF < 60) and high SHR (SHR > 0.99) in comparison to the other groups (log-rank *p* < 0.001). In adjusted analysis, after accounting for age, sex, diabetes, and NT-proBNP, elevated SHR remained independently predictive of adverse outcomes (adjusted HR: 2.34, 95% CI 1.49–3.67; *p* < 0.001). Furthermore, adding SHR to a model with MAGGIC score provided an incremental improvement in predicting adverse events. Additionally, SHR displayed a slight correlation with NT-proBNP.

**Conclusion:**

Elevated SHR was independently associated with an increased risk for composite events of all-cause, cardiovascular mortality, and HF readmission than those with lower SHR. SHR is a valuable tool for predicting and stratifying long-term adverse risks among HFpEF patients.

**Supplementary Information:**

The online version contains supplementary material available at 10.1186/s12933-024-02157-7.

## Introduction

Heart Failure (HF) with preserved ejection fraction (HFpEF) is a growing public health burden characterized by increased left ventricular (LV) filling pressures and impaired diastolic function [1]. Despite being a prevalent condition, the intricate interplay between various cardiac and non-cardiac factors contributing to HFpEF remains incompletely understood [2–4]. HFpEF persists as an unaddressed clinical demand, representing a multifaceted complex entity associated with a dire prognosis and substantial healthcare costs [5, 6]. Over 50% of these patients are at risk of rehospitalization and mortality within 5 years, demonstrating outcomes comparable to individuals with HF with reduced ejection fraction (HFrEF) [7]. Hence, there is a need for strategies to recognize high-risk individuals within the HFpEF patient population to prevent further decline and enhance overall well-being.

Emerging evidence suggests that metabolic disturbances, such as hyperglycemia may play a pivotal role in HFpEF [3, 8–10]. Stress hyperglycemia, a temporary rise in blood glucose levels due to physiological stressors, is often seen in acute clinical settings, including in patients with acute HF [11–14]. Elevated admission glucose levels, indicating stress hyperglycemia, independently predict adverse outcomes in individuals with potential cardiovascular diseases, including those with HFpEF [12, 15–17]. Nevertheless, stress hyperglycemia at admission can stem from either chronic hyperglycemia or an acute stress response and may not precisely indicate true acute glycemia. The stress hyperglycemia ratio (SHR), a new marker, estimates the true acute hyperglycemia state using acute admission glucose levels and background chronic glycemic values. Multiple studies have assessed its role across various cardiovascular diseases, indicating its potential as a predictor of future risk, regardless of diabetes presence [18–21]. While recent studies have explored the impact of SHR on short- and long-term outcomes in diabetic HF patients, there is no data on its specific impact on the long-term prognosis of HFpEF, encompassing both diabetic and non-diabetic individuals [22, 23]. Consequently, there is a need to clarify the potential role of SHR within clinical risk strategies for patients with HFpEF.

Hence, this study aims to examine the long-term effects of SHR for composite outcomes in individuals with HFpEF over a 41-month follow-up period and elucidate whether it can provide any clinical insights in the whole HFpEF population.

## Methods

### Study design and population

Participants referred to the Department of Cardiology at Shanghai 10th People’s Hospital were included in this observational-retrospective study. We enrolled individuals diagnosed with HFpEF between 2015 and 2023 based on the criteria established by the European Society of Cardiology for HF [1]. This diagnosis required the presence of HF signs/symptoms along with the following criteria: In brief, (1) preserved LVEF > 50% and (2) at least one major objective criterion for HF, i.e. raised levels of N-terminal pro-brain natriuretic peptide (NT-proBNP) and presence of cardiac functional and structural abnormalities as identified by echocardiography. Exclusion criteria encompassed patients < 18 years old, those with severe liver or kidney disease, significant valvular disease, a history of myocardial infarction, pulmonary disease, and obstructive coronary artery disease (stenosis > 50%) detected during angiography. Patients with missing SHR information and follow-up data were also excluded (Additional file 1: Figure S1). The study followed the Declaration of Helsinki principles, and all participants provided written informed consent. Study approval was granted by the Ethics Board of Shanghai Tenth People’s Hospital (Protocol No. 23K107).

### Data collection

Comprehensive information for demographic data, baseline characteristics, comorbidities, such as sex, age, New York Heart Association (NYHA) class, body mass index (BMI), diabetes history, medication, laboratory, angiography, and echocardiography performed during admission were extracted from our centers’ electronic system database. Routine hematological and biochemical parameters, such as hemoglobin, fasting blood glucose, HbA1c, NT-proBNP, estimated glomerular filtration rate (eGFR), low-density lipoprotein cholesterol (LDL), thyroid stimulating hormone (TSH), C-reactive protein (CRP), and other biomarkers assessed through venous blood samples drawn from the cubital vein within the initial 24 h of admission, following a fasting period of at least 8 h. The analysis of blood glucose and biochemical parameters was conducted using Abbott Laboratories instrument (Chicago, US). Comprehensive echocardiography with tissue doppler imaging was performed by board-certified cardiologists according to recommended standards [24].

### Assessment of SHR

The admission blood glucose (ABG) refers to the fasting blood glucose levels measured within the initial 24 h of hospital admission. SHR is computed by the formula: SHR = ABG (mmol/L)/ [1.59×HbA1c (%)-2.59] [18]. The optimal cut-off for SHR in predicting long-term events was identified as 0.99, determined through the area under the curve (AUC) in the receiver-operator characteristic (ROC), and is derived from Youden index. After determining the optimal SHR cut-off, patients were stratified into two groups; 124 patients were classified into the high SHR group (> 0.99), while 276 patients comprised the low SHR group (SHR ≤ 0.99).

### Outcomes

The study participants were monitored for an average of 41 months to evaluate a composite outcome encompassing all-cause mortality (death resulting from any reason, encompassing cardiovascular reasons) and readmission for HF (hospitalization specifically for HF, necessitating an escalation in treatment). Two specialists, unbiased to the study, documented the follow-up information by examining the medical case files and conducting telephonic discussions.

### Statistical analysis

The categorical variables are shown in percentages, while continuous variables are shown using either the mean ± standard deviation or the median (interquartile range). Baseline characteristics are compared between groups employing independent-sample t-tests, Mann-Whitney U tests, or Pearson’s chi-square as appropriate. Kaplan-Meier analysis was employed to assess the incidence rate of composite events and the influence of SHR, with differences compared by a log-rank test. To quantify the linear relationship between SHR and parameters reflecting HF severity, Pearson’s correlation coefficient (r) was employed. The Cox analysis is employed to evaluate the hazard ratios (HRs) with 95% confidence intervals (CIs) for composite outcomes and to identify the univariate and multivariate predictive factors. Essential and pivotal cardiovascular risk factors, clinically recognized for their role in facilitating the future risk of adverse outcomes in patients with HFpEF [25–27], including age, NYHA class, BMI, sex, atrial fibrillation (AF), hypertension, smoking, diabetes, chronic kidney disease (CKD), echocardiography values, and pertinent laboratory variables were imputed into the univariate analysis. Variables that attain statistical significance with a significance level of *P* < 0.10 in univariate testing are subsequently inserted in the multivariate model. The predictive value and optimal cut-off for SHR in predicting events were assessed using the AUC in the ROC and is calculated from the Youden index when sensitivity and specificity correspond to the maximum. Moreover, we assessed the predictive performance of SHR for composite events by incorporating it into the Meta-Analysis Global Group in Chronic HF (MAGGIC) risk model with clinical risk factors, using ROC analysis, and calculated the AUC. The incremental prognostic value of SHR was further assessed by the integrated discrimination improvement (IDI) and the net reclassification improvement (NRI) [28]. Statistical significance is determined when p-value < 0.05, and all tests are conducted with a 2-sided approach. The data is analyzed through SPSS (version; 24.0), while the GraphPad software (version; 8.0.1) was utilized for figure generation.

## Results

### Baseline characteristics

A total of 3060 patients admitted to the hospital for acute HF were consecutively recruited. Following the application of exclusion criteria, 400 patients with HFpEF were ultimately included in the final analysis for this study, of whom 56.5% were female, and had an average age of 71.0 ± 7.8 years.

Compared to patients without events, those with events were older and exhibited significantly elevated levels of SHR (0.94 ± 0.3 vs. 0.83 ± 0.2, *p* < 0.001) (Additional file 1: Table S1). The scatterplot of SHR across the two groups is shown in the Additional file 1: Figure S 2.

Subsequently, all patients were classified into two groups (high SHR > 0.99 and low SHR ≤ 0.99) based on the optimal cut-off from ROC analysis and Youden index, where sensitivity and specificity reached their maximum values (AUC:0.62; 95%CI; 0.56–0.67; p < 0.001, specificity: 0.82, sensitivity: 0.46). The distribution of baseline characteristics between the two groups (high and low SHR) is presented in Table 1. Individuals with a high SHR exhibited a higher burden of comorbidities, including AF and CKD, along with an elevated heart rate and a higher prevalence of NYHA class III-IV compared to those with a low SHR. Additionally, patients with high SHR had significantly elevated levels of NT-proBNP, serum creatinine, LDL cholesterol, CRP, and troponin, along with lower hemoglobin levels than those with low SHR. In echocardiography data, patients with a high SHR had larger left ventricular end-diastolic diameter (LVEDD), left ventricular end-diastolic diameter (LVESD), and a lower LVEF, compared to those with low SHR. Furthermore, parameters reflecting diastolic function, such as septal E/e’ was higher and e’ was lower, in the high SHR group. Medications, including beta blockers, mineral corticoid receptor antagonists (MCRA), and angiotensin-converting enzyme inhibitors/angiotensin receptor blockers (ACE + ARB), showed comparable distribution between the two groups except that diuretic use was more prevalent in the high SHR group.

**Table 1:** Baseline characteristics of study population according to SHR cut-off

	All patients (*n* = 400)	Patients with SHR > 0.99 (*n* = 124)	Patients with SHR ≤ 0.99 (*n* = 276)	P value
**Baseline variables**				
Age (years)	71.0 ± 7.8	71.1 ± 8.3	70.9 ± 7.6	0.896
Female, n (%)	226 (56.5)	67 (54.0)	159 (57.6)	0.505
BMI (kg/m2)	25.2 ± 3.8	25.2 ± 4.3	25.2 ± 3.6	0.989
NYHA class III-IV, n (%)	201 (50.3)	73 (58.9)	128 (46.4)	0.021
Systolic BP (mmHg)	140.9 ± 23.2	137.8 ± 24.2	142.3 ± 22.6	0.074
Diastolic BP (mmHg)	77.9 ± 13.5	77.3 ± 14.5	78.2 ± 13.0	0.538
Heart rate	80.8 ± 17.2	83.8 ± 19.6	79.5 ± 15.9	0.022
**Comorbidities, n (%)**				
CHD	162 (40.5)	52 (41.9)	110 (39.9)	0.695
Atrial fibrillation	103 (25.8)	40 (32.3)	63 (22.8)	0.046
Alcohol	43 (10.8)	16 (12.9)	27 (9.8)	0.351
Smoking	86 (21.5)	34 (27.4)	52 (18.8)	0.053
Hypertension	298 (74.5)	86 (69.4)	212 (76.8)	0.114
Diabetes	166 (41.5)	58 (46.8)	108 (39.1)	0.151
Chronic Kidney Disease	52 (13.0)	24 (19.4)	28 (10.1)	0.011
Hyperlipidemia	124 (31.0)	46 (37.1)	78 (28.3)	0.077
**Laboratory data**				
HbA1c (g/L) (%)	6.6 ± 1.3	6.7 ± 1.5	6.5 ± 1.1	0.059
ABG (µmol/L)	6.9 ± 2.7	9.4 ± 3.0	5.8 ± 1.5	< 0.001
Haemoglobin, g/dL	126.9 ± 19.1	123.0 ± 22.3	128.7 ± 17.3	0.005
TSH (mg/l)	3.8 ± 9.0	4.1 ± 10.4	3.8 ± 8.4	0.795
ALT	26.5 ± 24.9	28.4 ± 20.6	25.6 ± 26.6	0.297
Troponin T	0.06 ± 0.1	0.08 ± 0.1	0.05 ± 0.1	0.032
NT-proBNP (pg/mL)	1127.0 (573.8-2216.3)	1476.5 (774.5-2985.5)	946.0 (485-2010.5)	< 0.001
LDL	2.1 ± 0.8	2.1 ± 0.8	2.1 ± 0.8	0.463
Total cholesterol (mmol/L)	3.9 ± 1.1	4.0 ± 1.2	3.9 ± 1.1	0.386
Creatinine (mg/dl)	90.3 ± 39.6	98.7 ± 48.3	86.7 ± 34.7	0.006
eGFR	70.5 ± 23.5	68.9 ± 22.2	71.2 ± 24.1	0.357
Blood urea	7.6 ± 6.4	8.6 ± 5.4	7.1 ± 6.7	0.036
Potassium (mmol/L)	4.0 ± 0.6	3.9 ± 0.7	4.0 ± 0.6	0.286
C-reactive protein	3.3 (3.0-6.3)	4.7 (3.1–8.7)	3.2 (3.0-5.8)	0.001
**Medication data**				
Beta-blockers	242 (60.5)	76 (61.3)	166 (60.1)	0.828
Diuretics	192 (48.0)	70 (56.5)	122 (44.2)	0.023
MCRA	129 (32.3)	45 (36.3)	84 (30.4)	0.247
Calcium channel blockers	124 (31.0)	32 (25.8)	92 (33.3)	0.132
ACEI + ARB	197 (49.3)	58 (46.8)	139 (50.4)	0.507
Statins	335 (83.8)	99 (79.8)	236 (85.5)	0.155
**Echocardiography**				
LAVI, mL/m2	42.6 ± 6.2	43.3 ± 6.4	42.3 ± 6.0	0.149
LVEDD (mm)	47.4 ± 5.6	48.2 ± 5.6	47.0 ± 5.6	0.038
LVESD (mm)	31.8 ± 6.8	33.4 ± 7.9	31.1 ± 6.1	0.002
LVEF (%)	60.5 ± 5.0	59.6 ± 4.6	60.9 ± 5.1	0.015
e’, cm/s	7.0 (6.0–8.0)	6.0 (5.0–8.0)	7.0 (6.0–8.0)	0.020
Septal E/e’	15.0 ± 3.0	15.6 ± 3.4	14.7 ± 2.8	0.006
PASP	38.7 ± 11.4	39.5 ± 13.3	38.3 ± 10.4	0.333

SHR: stress hyperglycemia ratio; BMI: body mass index; NYHA class: New York heart association; BP: blood pressure; CHD: coronary heart disease; HbA1c: glycated hemoglobin; ABG: admission blood glucose; TSH: thyroid stimulating hormone; ALT: alanine aminotransferase; NT-proBNP: N-terminal pro–B-type natriuretic peptide; LDL: low-density lipoprotein; eGFR: estimated glomerular filtration rate; MCRA: mineralocorticoid receptor antagonist; ACEI + ARB: angiotensin-converting enzyme inhibitors/angiotensin receptor blockers; LAVI: left atrial volume index; LVEDD: left ventricular end-diastolic diameter; LVESD: left ventricular end-systolic diameter; LVEF: left ventricular ejection fraction; e’: peak LV velocity; E/e’: mean septal velocity; PASP: pulmonary artery systolic pressure

### Relationship between SHR and HF severity parameters

Additional file 1: Figure S3. shows the relationship between SHR and markers of HF severity. The correlation between SHR and NTproBNP values, SHR and LV early filling pressure e’, and SHR and E/e’ is investigated. SHR showed a weak positive correlation with NT-proBNP (r = 0.134, p = 0.007) and E/e’ (r = 0.131, p = 0.010). While, negative correlations were identified between SHR and e’ (*r* = − 0.123, *p* = 0.034).

### SHR and clinical outcomes

**Fig. 1:**
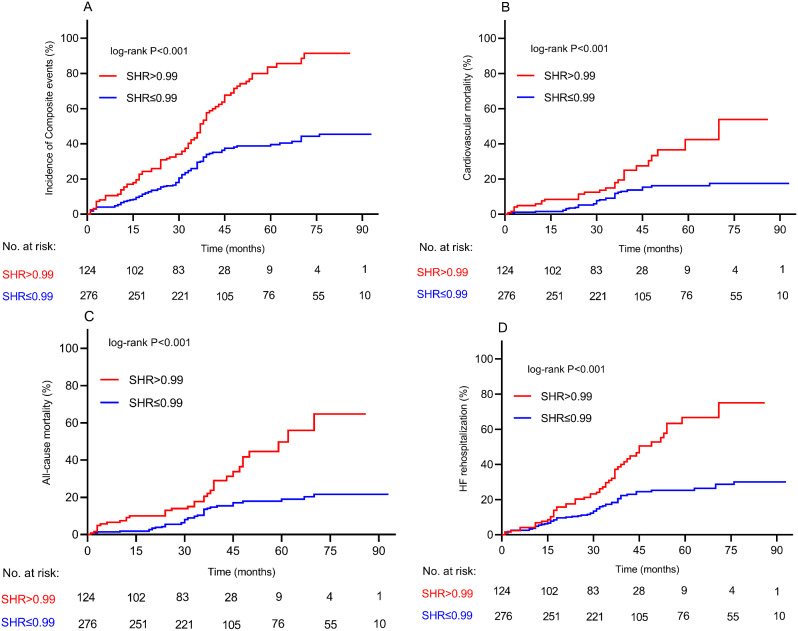
**(A)** Kaplan-Meier survival curves for incidence of composite events stratified by SHR cutoff among HFpEF patients; **(B)** Incidence of cardiovascular mortality **(C)** Incidence of All-cause mortality **(D)** Incidence of HF rehospitalization SHR: stress hyperglycemia ratio; HF: heart failure

**Fig. 2:**
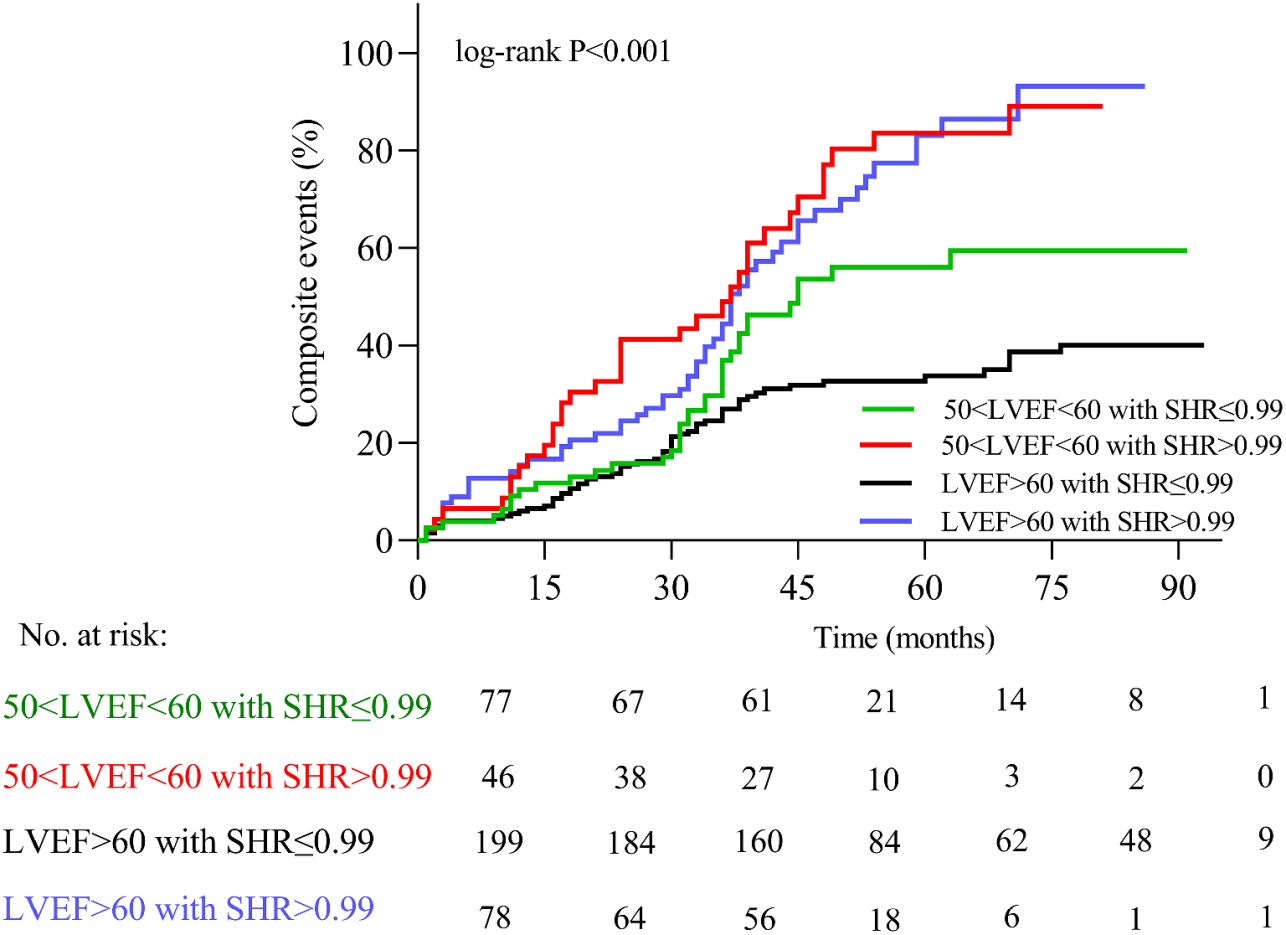
Estimates of composite events stratified by LVEF (50 < LVEF < 60 and > 60) and SHR (SHR ≤ 0.99 and SHR > 0.99). LVEF: left ventricular ejection fraction; SHR: stress hyperglycemia ratio

In the present study, over the follow-up (mean, 41.4 months), 190 patients (47.5%) experienced composite events of all-cause mortality (75/18.8), including cardiovascular mortality (62/15.5) and HF readmissions (115/28.8). The high SHR group was associated with a significantly higher risk of composite events as compared to low SHR group (71.0% vs. 37.0%, *p* < 0.001) (Additional file 1: Table S2). Additionally, Kaplan–Meier survival analyses results further demonstrated that patients in high SHR group were associated with a worse overall survival (log-rank, *p* < 0.001) (Fig. 1 A). Similarly, we observed consistent results when the high SHR group was tested against individual endpoints (cardiovascular mortality, all-cause mortality, and HF readmission, all *p* < 0.05) (Fig. 1. B, C, D). Upon subgroup analysis, the study cohort was stratified into four subgroups based on LVEF and SHR levels: 50 < LVEF < 60 with SHR ≤ 0.99, 50 < LVEF < 60 with SHR > 0.99, LVEF > 60 with SHR ≤ 0.99, and LVEF > 60 with SHR > 0.99. The findings indicated a notably poorer prognosis in patients with 50 < LVEF < 60 and SHR > 0.99 in comparison to the other groups (log-rank *p* < 0.001) (Fig. 2). Moreover, subgroup analyses were performed to examine the effects of SHR on outcomes based on the tertiles of SHR (SHR1: ≤0.74, SHR2: >0.74-≤0.98, and SHR3: >0.98). These analyses showed that the group with the highest SHR tertile (SHR3) encountered the most severe adverse events. Conversely, the group with the lowest SHR tertile (SHR1) also displayed a higher incidence of adverse events compared to the SHR2 group, as evidenced by the log-rank test (*p* < 0.001). (Additional file 1: Figure S4).

### Prognostic value of SHR

The Cox analyses, including predictive factors of clinical outcomes in both univariate and multivariate contexts, are documented in Table 2. Notably, SHR > 0.99 (HR:2.57,p < 0.001), advanced age (HR:1.04,p = 0.001), sex (HR = 1.44, p = 0.013), BMI (HR:1.04,p = 0.036), diabetes (HR = 1.45,p = 0.011), AF (HR:1.62,p = 0.002), smoking (HR:1.36,p = 0.062), CKD (HR:1.44,p = 0.076), elevated NT-proBNP (HR:1.00, p = 0.001), LAVI (HR:1.03,p = 0.028), LVEF (HR:0.96,p = 0.007), e’ (HR:0.87,p = 0.047), E/e’ (HR:1.06,*p* = 0.030), eGFR (HR:0.99,*p* = 0.001), CRP (HR:1.03,*p* = 0.010) and the use of calcium channel blockers (HR:0.73,*p* = 0.054), ACE/ARB (HR:0.73,*p* = 0.032) and statins (HR:0.74,*p* = 0.099), were identified as independent predictive factors of adverse outcomes through univariate analysis. In the multivariate analysis, after adjusting for potential confounders, the SHR > 0.99 (adjusted HR:2.34, *p* < 0.001) remained a significant predictor of long-term events along with age, sex, diabetes, NT-proBNP, and the use of ACE/ARB.

**Table 2:** Univariate and Multivariate Cox regression analysis for clinical events

	Univariate		Multivariate	
	HR (95% CI)	P value	HR (95% CI)	P value
Age	1.04 (1.01–1.06)	0.001	1.04 (1.01–1.08)	0.018
NYHA	1.16 (0.87–1.54)	0.310		
Sex	1.44 (1.08–1.93)	0.013	1.87 (1.07–3.27)	0.027
Smoking	1.36 (0.98–1.89)	0.062	1.47 (0.88–2.47)	0.144
BMI	1.04 (1.00-1.08)	0.036	1.00 (0.95–1.06)	0.947
Atrial fibrillation	1.62 (1.20–2.18)	0.002	0.92 (0.52–1.62)	0.765
Hypertension	0.92 (0.66–1.28)	0.630		
Chronic kidney disease	1.44 (0.96–2.15)	0.076	1.13 (0.60–2.12)	0.704
Hyperlipidemia	1.08 (0.80–1.45)	0.610		
CHD	0.91 (0.68–1.22)	0.521		
Diabetes	1.45 (1.09–1.92)	0.011	1.67 (1.07–2.61)	0.024
LDL	1.07 (0.91–1.27)	0.409		
Total cholesterol	0.94 (0.83–1.06)	0.315		
eGFR	0.99(0.98-1.00)	0.001	0.99 (0.98-1.00)	0.132
C-reactive protein	1.03 (1.01–1.06)	0.010	1.04 (0.99–1.07)	0.056
NT-proBNP	1.00 (1.00–1.00)	0.001	1.00 (1.00–1.00)	0.014
LVEF	0.96 (0.93–0.99)	0.007	1.01 (0.96–1.05)	0.736
LAVI	1.03 (1.00-1.05)	0.028	1.04 (0.99–1.11)	0.097
e’	0.87 (0.76–0.99)	0.047	0.86 (0.72–1.03)	0.110
E/e’	1.06 (1.01–1.11)	0.030	1.04 (0.97–1.12)	0.212
PASP	1.01 (1.00-1.03)	0.100		
LVEDD	1.02 (1.00-1.05)	0.059	0.98 (0.93–1.03)	0.558
Beta-blockers	0.89 (0.67–1.19)	0.445		
ACE + ARB	0.73 (0.55–0.97)	0.032	0.63 (0.39–0.99)	0.048
Diuretic	0.93 (0.70–1.24)	0.619		
MCRA	0.99 (0.74–1.34)	0.968		
Statins	0.74 (0.52–1.06)	0.099	0.59 (0.32–1.08)	0.089
Calcium channel blockers	0.73 (0.53–1.01)	0.054	0.80 (0.49–1.32)	0.382
Anticoagulant	1.28 (0.89–1.85)	0.180		
SHR > 0.99	2.57 (1.93–3.44)	< 0.001	2.34 (1.49–3.67)	< 0.001

NYHA: New York heart association; BMI: body mass index; CHD: coronary heart disease; LDL: low-density lipoprotein; eGFR: estimated glomerular filtration rate; NT-proBNP: N-terminal pro–B-type natriuretic peptide; LVEF: left ventricular ejection fractions; LAVI: left atrial volume index; e’: peak LV velocity; E/e’: mean septal velocity; PASP: pulmonary artery systolic pressure; LVEDD: left ventricular end-diastolic dimension; ACE + ARB: angiotensin-converting enzyme inhibitor/angiotensin receptor blocker; MCRA: mineralocorticoid receptor antagonist; SHR: stress hyperglycemia ratio; HR: hazard ratio; CI: confidence interval

### Predictive accuracy of SHR for composite events

**Fig. 3:**
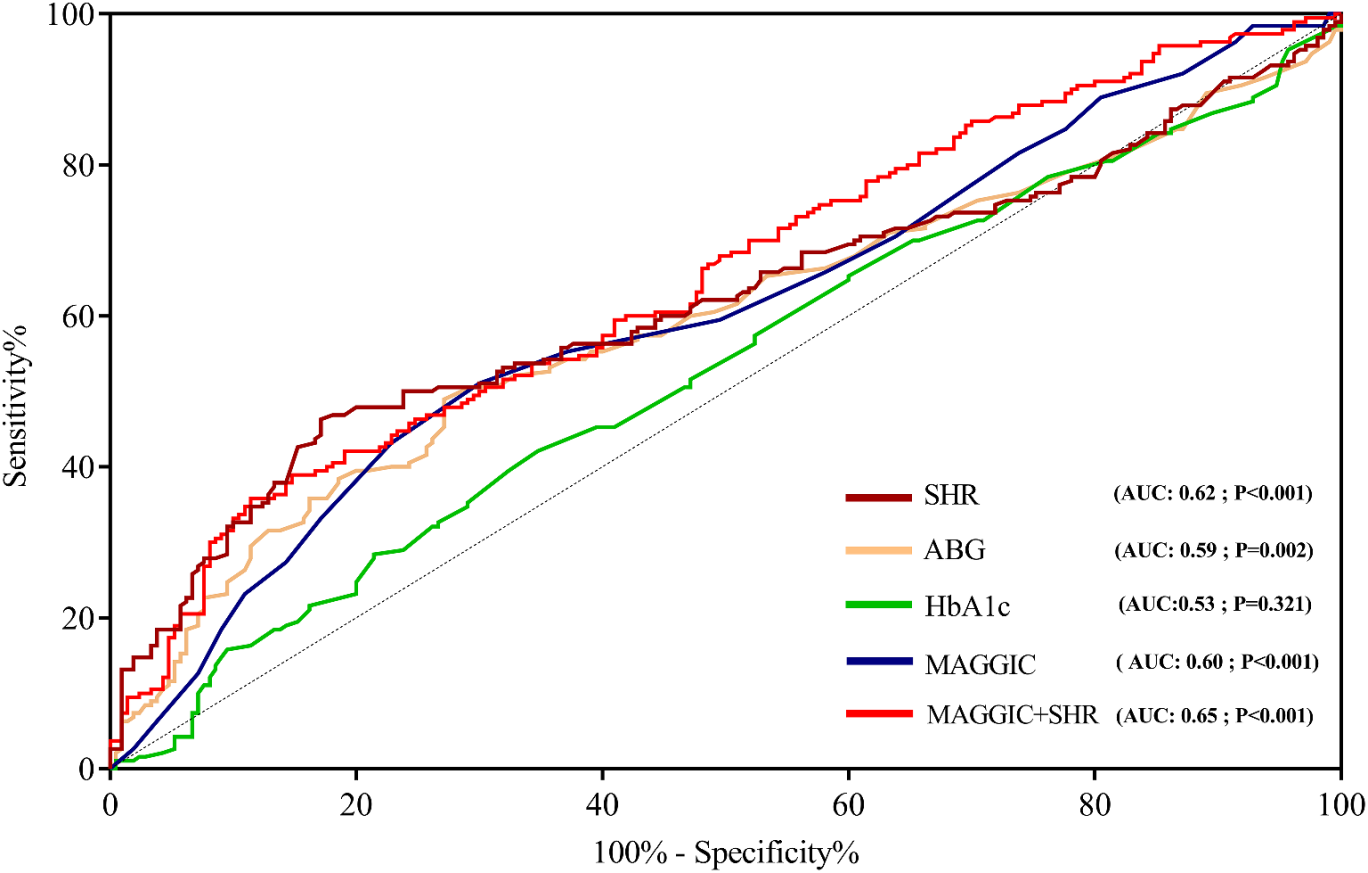
Receiver operating curves of SHR, stress hyperglycemia, and MAGGIC score for predicting adverse events AUC: area under the curve; SHR: stress hyperglycemia ratio; ABG: admission blood glucose; HbA1c: glycated hemoglobin; MAGGIC risk score; meta-analysis global group in chronic heart failure

The prognostic accuracy of SHR, ABG, and HbA1c is depicted in Fig. 3. SHR, representing the combined ratio of ABG/HbA1c levels, was compared with stress hyperglycemia (ABG alone) and HbA1c. The findings demonstrated that SHR (AUC: 0.62; 95% CI: 0.56–0.67; *p* < 0.001) exhibited superior predictive ability for composite events compared to stress hyperglycemia (AUC: 0.59; 95% CI: 0.54–0.65; *p* = 0.002) or HbA1c (AUC: 0.53; 95% CI: 0.47–0.59; *p* = 0.321) (Fig. 3).

### Incremental value of SHR for predicting composite events

We further assessed the incremental value of SHR for composite events by incorporating it into the established HF risk score, the MAGGIC score (AUC: 0.60; 95% CI: 0.55–0.66; *p* < 0.001). The AUC was calculated for the combination of MAGGIC and SHR. The results indicated that the addition of SHR to the validated MAGGIC risk model slightly improved predictive accuracy for composite events (AUC: 0.65; 95% CI: 0.59–0.70; *p* < 0.001). This enhancement was substantiated by an increase in the IDI (0.04, 95% CI: 0.02–0.06, *p* = 0.044) and NRI (0.41, 95% CI: 0.22–0.60, *p* = 0.027) as illustrated in Fig. 3; Table 3.

**Table 3:** Model improvement for MAGGIC risk score in combination with SHR

Models	AUC (95% CI)	p value	cNRI (95% CI)	p value	IDI (95% CI)	p value
Model 1	0.60 (0.55–0.66)	< 0.001	Reference		Reference	
Model 2	0.65 (0.59–0.70)	< 0.001	0.41 (0.22–0.60)	0.027	0.04 (0.02–0.06)	0.044

Model 1 = MAGGIC risk score; Model 2 = MAGGIC risk score + SHR

AUC: area under curve; IDI: integrated discrimination improvement; cNRI: continuous net reclassification index; CI: confidence interval; MAGGIC risk score: meta-analysis global group in chronic heart failure; SHR: stress hyperglycemia ratio

## Discussion

The findings of this study reveal potential associations between the SHR and long-term outcomes in HFpEF patients. We observed that elevated SHR was associated with a heightened risk of composite events and an optimal cut-off for risk prediction was SHR > 0.99. In addition, SHR demonstrated a slight correlation with key parameters indicating the HF severity, such as NT-proBNP, septal E/e’ ratio, and e’, highlighting its potential role as a prognostic indicator in this patient population.

HFpEF constitutes a significant portion of cases within the HF spectrum, with various studies highlighting its detrimental effects and severe short- and long-term prognosis [1]. HF registries across diverse regions among patients with HFpEF indicated an all-cause mortality and HF rehospitalization rate for 1-year ranging between 19 and 29%, whereas, the 5-year rate was as high as 75% [7, 25, 29]. Furthermore, in a recent 10-year prospective, multicenter study involving HFpEF individuals, the mortality rates at 1, 3, 5, and 10 years were 15%, 31%, 47%, and 74%, while the composite rates were 35%, 54%, 67%, and 84%, respectively [30]. In our study, after an average follow-up of 41-months, the composite event rate was 47.5%, aligning with earlier research, emphasizing the heightened burden of this entity on public health. Varied LVEF criteria in defining HFpEF, study design, and population characteristics may explain varying clinical event rates across HFpEF studies. Identifying additional risk factors in HFpEF is crucial to enhance risk stratification, tailor interventions, and improve outcomes.

Stress hyperglycemia is often seen in HF patients and is potentially associated with an elevated risk of adverse events and deteriorating cardiovascular outcomes [14, 15]. Stress hyperglycemia is linked to inflammation, oxidative stress, microvascular injury, endothelial dysfunction, and prothrombotic state, contributing to impaired myocardial blood flow, and diminished cardiac function [31]. In HFpEF patients, stress hyperglycemia was identified as a robust predictor of all-cause and cardiac mortality, specifically in those without diabetes [17]. While stress hyperglycemia may not potentially distinguish among diverse causes of absolute hyperglycemia, SHR is a combined measure of a patient’s acute-phase and chronic glycemia values, it reflects the true stress hyperglycemia state and has been found to be a potential determinant of adverse risk in multiple cardiovascular diseases [32]. Zhou et al. found that both high and low SHR indicate a poor prognosis in patients with HF and type 2 diabetes during hospitalization [22]. In a recent study, a U-shaped association was identified between the SHR and all-cause, cardiac death, and HF readmission in individuals with acute decompensated HF and diabetes after a 3-year follow-up. These authors in their subgroup analysis further showed that this association persisted even in diabetic patients with HFpEF [23]. While previous studies did not specify HF type [22], and primarily focused on diabetic patients [23], SHR is recognized to impact prognosis irrespective of diabetes status [18–20]. However, the prognostic role of SHR encompassing the whole HFpEF population has yet to be explored. The effectiveness of using SHR as an indicator for anticipating future risk in diabetic HF patients raises the question of its predictive applicability in the broader HFpEF population, encompassing both diabetic and non-diabetic individuals. In line with these findings, our study also established that HFpEF patients with a high SHR are at significantly greater risk of composite events. In adjusted analysis, after accounting for confounders, a high SHR remained independently predictive of worse survival after long-term follow-up. Furthermore, the inclusion of SHR to the validated MAGGIC risk model enhanced predictive accuracy for composite events. Additionally, on examining the effects of SHR on outcomes across tertiles, we noted that individuals in the highest SHR tertile (SHR3) experienced the most severe adverse events. Conversely, the group in the lowest SHR tertile (SHR1) also exhibited a higher incidence of events compared to the SHR2 group, suggesting a potential association between both low and high SHR levels and a worsened prognosis. Thus, our study provides meaningful evidence confirming the applicability of SHR and its prognostic significance across the broader HFpEF population, regardless of diabetes status. Notably, our findings indicate no interaction effect of SHR on patients with diabetes compared to those without diabetes in HFpEF, indicating SHR may affect both diabetic and non-diabetic HFpEF patients (interaction *p* = 0.114, data not shown).

Several studies have proposed that stress hyperglycemia may indicate the severity of the disease, revealing a potential correlation with various relevant factors, including vascular inflammation, worsening LV geometry, myocardial remodeling, and damage [33–35]. Our findings revealed that elevated SHR levels were associated with worse functioning status and unfavorable baseline characteristics, and patients with high SHR demonstrated a higher prevalence of comorbidities, including AF, CKD, and NYHA class III-IV. The high SHR group exhibited elevated levels of NT-proBNP, and serum creatinine, along with lower levels of hemoglobin and eGFR, as well as worse cardiac functional parameters in comparison to patients with lower SHR levels. Furthermore, we observed a correlation between SHR and markers of diastolic dysfunction such as E/e’, and e’. Interestingly, we found SHR correlated with NT-proBNP, which is a marker of HF severity. This association of high SHR with elevated NT-proBNP may reflect significant myocardial disease. Furthermore, our study revealed that patients with high SHR and low LVEF had a worse prognosis compared to other groups. This observation is supported by several studies where SHR was correlated with worse LV mechanics, myocardial damage, and disease severity [18, 22, 36]. This evidence implies that elevated SHR might play a pivotal role in the core pathological mechanisms of HFpEF, potentially serving as an indicator of disease severity.

The underlying mechanisms of HFpEF are multifactorial, encompassing systemic inflammation, endothelial dysfunction, myocardial stiffening, and diastolic dysfunction [3]. In HFpEF, comorbidities, including diabetes, obesity, AF, and CKD, drive a chronic low-grade inflammatory state, impairing vasodilation and causing cardiomyocyte injury and endothelial dysfunction. Elevated glucose levels intensify these effects, triggering inflammatory and fibrotic pathways, increasing extracellular matrix secretion, and contributing to myocardial stiffness. The persistent microvascular injury, driven by impaired vasodilation, pro-inflammatory processes, aggravated myocardial damage as seen in elevated SHR reflecting stress hyperglycemia, elevated filling pressures, and compromised contractility and perfusion, may create a ‘perfect storm’ for the development of myocardial fibrosis which is central to HFpEF [22, 23, 37, 38]. This convergence could contribute to the exacerbation of HF and adverse outcomes.

The identification of an optimal SHR cut-off value for distinguishing high-risk patients varies among studies, and is primarily influenced by the diversity in risk factors and pathophysiological mechanisms specific to each disease. In the investigation involving diabetic patients with HF, a SHR value of < 0.78 and > 1.09 demonstrated predictive capacity for cardiac, kidney, and infection events during hospitalization [22]. Additionally, Zhou et al. [23] described SHR values < 0.64 and > 1.14 were indicative of heightened risk in diabetic HF patients after a follow-up of 3-years. In a study conducted by Cui et al. among acute myocardial infarction patients, SHR cutoff values of 1.20 and 1.08 effectively predicted long-term mortality [19]. In patients with MINOCA, a cutoff value of 0.78 demonstrated predictive ability for adverse events during a long-term follow-up [18]. In the present study, we identified a cut-off value of 0.99 was predictive of adverse outcomes and was able to discriminate high-risk patients in whole the HFpEF population. The determination of a definitive SHR cut-off value for distinguishing high-risk patients prone to developing adverse events in HFpEF has not been thoroughly examined and requires further investigation.

Overall, our findings contribute valuable insights to existing knowledge, suggesting potential implications for clinical decision-making. The data propose that early intervention targeting SHR in HFpEF may mitigate future risks and enhance prognosis. However, further prospective studies are essential to validate and elaborate on these observations.

### Limitations

Our study has several limitations that warrant consideration. First, its observational and retrospective design implies that the results should be interpreted as hypothesis-generating rather than definitive. Second, being conducted at a single center with a relatively small sample size and a short follow-up duration might introduce limitations to generalizability. Despite adjusting for potential confounders, the possibility of unmeasured factors, influencing the results cannot be entirely excluded. Additionally, the lack of information on other inflammatory markers, the use of hypoglycemic drugs, and serial changes in blood glucose/glycated hemoglobin during the hospitalization and subsequent follow-up period could impact long-term outcomes, limiting our comprehensive understanding of poor prognosis. Due to pre-specified clinical endpoints, we didn’t assess other unspecified outcomes like new-onset AF in relation to SHR and primary endpoints. Finally, the observational nature of our study design precludes the identification of causal mechanisms, additional research is essential to elucidate the mechanisms contributing to the deterioration of outcomes in HFpEF and its association with SHR. Further investigations are warranted to assess whether interventions targeting these mechanisms can enhance survival and improve outcomes in HFpEF patients.

## Conclusion

SHR demonstrated a significant association with long-term outcomes in the entire HFpEF population. Elevated SHR is an independent predictor for poor survival in patients with HFpEF and seems to correlate with the marker of HF severity. These findings suggest that SHR could offer valuable insights for effective risk stratification in this patient population.

### Electronic supplementary material

Below is the link to the electronic supplementary material.

**Additional file 1:** **Table S1**. Baseline characteristics of study population stratified by events vs non-events. **Table S2**. Outcomes of study population according to SHR cut-off. **Figure S1**. Flowchart of patients enrolled. **Figure S2**. The distribution of SHR among HFpEF patients with and without events. **Figure S3**. Relationship between SHR and HF parameters

## Data Availability

The dataset examined in this study is available upon reasonable request from the corresponding author.

## Abbreviations

HF: heart failure
HFpEF: heart failure with preserved ejection fraction
SHR: stress hyperglycemia ratio
NYHA class: New York Heart Association
CHD: coronary heart disease
AF: atrial fibrillation
BMI: body mass index
CKD: chronic kidney disease
LAVI: left atrial volume index
LVEDD: left ventricular end-diastolic diameter
LVEF: left ventricular ejection fractions
e’: peak LV velocity
E/e’: mean septal velocity
PASP: pulmonary artery systolic pressure
ABG: admission blood glucose
HbA1c: glycated hemoglobin
LDL-cholesterol: low-density lipoprotein-cholesterol
ALT: alanine aminotransferase
eGFR: estimated glomerular filtration rate
HR: hazard ratio
BP: blood pressure
NT-proBNP: N-terminal pro-brain natriuretic peptide
MCRA: mineralocorticoid receptor antagonist
ACEI + ARB: angiotensin-converting enzyme inhibitors/angiotensin receptor blockers
