# Supplementary material for: Stress hyperglycemia ratio as a prognostic indicator for long-term adverse outcomes in heart failure with preserved ejection fraction
Source: Cardiovasc Diabetol. 2024 Feb 13;23:67. doi: 10.1186/s12933-024-02157-7 (PMC10865536; doi:10.1186/s12933-024-02157-7)
Supplement: Supplementary file 1 — Additional file 1: Table S1. Baseline characteristics of study population stratified by events vs non-events. Table S2. Outcomes of study population according to SHR cut-off. Figure S1. Flowchart of patients enrolled. Figure S2. The distribution of SHR among HFpEF patients with and without events. Figure S3. Relationship between SHR and HF parameters [file 12933_2024_2157_MOESM1_ESM.docx]

**Table S1.** Baseline characteristics, laboratory, medications, and echocardiography data of the study population.

|  | All patients  (n=400) | HFpEF with  events (n=190) | HFpEF without  events (n=210) | P value |
| --- | --- | --- | --- | --- |
| Age (years) | 71.0±7.8 | 71.9±8.2 | 70.1±7.4 | 0.019 |
| Female, n (%) | 226 (56.5) | 106 (55.8) | 120 (57.1) | 0.785 |
| BMI (kg/m2) | 25.2±3.8 | 25.7±4.2 | 24.7±3.3 | 0.014 |
| NYHA class III-IV, n (%) | 201 (50.3) | 102 (53.7) | 99 (47.1) | 0.191 |
| Systolic BP (mmHg) | 140.9±23.2 | 139.2±24.5 | 142.4±21.9 | 0.160 |
| Diastolic BP (mmHg) | 77.9±13.5 | 76.5±14.6 | 79.2±12.2 | 0.044 |
| Heart rate (beats per minute) | 80.8±17.2 | 81.8±19.3 | 79.9±15.1 | 0.272 |
| **Comorbidities**, n (%) |  |  |  |  |
| CHD | 162 (40.5) | 75 (39.5) | 87 (41.4) | 0.691 |
| Atrial fibrillation | 103 (25.8) | 67 (35.3) | 36 (17.1) | 0.001 |
| Smoking | 86 (21.5) | 49 (25.8) | 37 (17.6) | 0.047 |
| Alcohol | 43 (10.8) | 24 (12.6) | 19 (9.0) | 0.248 |
| Hypertension | 298 (74.5) | 143 (75.3) | 155 (73.8) | 0.739 |
| Diabetes | 166 (41.5) | 95 (50.0) | 71 (33.8) | 0.001 |
| Hyperlipidemia | 124 (31.0) | 69 (36.3) | 55 (26.2) | 0.029 |
| Chronic Kidney Disease | 52 (13.0) | 28 (14.7) | 24 (11.4) | 0.326 |
| **Laboratory data** |  |  |  |  |
| HbA1c (g/L) (%) | 6.6±1.3 | 6.6±1.3 | 6.5±1.2 | 0.459 |
| ABG (μmol/L) | 6.9±2.7 | 7.4±3.1 | 6.4±2.2 | <0.001 |
| SHR | 0.88±0.2 | 0.94±0.3 | 0.83±0.2 | <0.001 |
| Haemoglobin, g/dL | 126.9±19.1 | 124.6±20.8 | 129.1±17.3 | 0.021 |
| TSH (mg/l) | 3.8±9.0 | 4.2±10.9 | 3.5±6.6 | 0.445 |
| ALT | 26.5±24.9 | 25.9±22.9 | 27.01±26.6 | 0.662 |
| Troponin T | 0.06±0.1 | 0.06±0.1 | 0.05±0.1 | 0.576 |
| NT-proBNP (pg/mL) | 1127.0 (573.8-2216.3) | 1295.0 (702.0-2747.5) | 878.5 (440.5-1803.3) | 0.001 |
| LDL | 2.1±0.8 | 2.2±0.9 | 2.0±0.8 | 0.056 |
| Total cholesterol | 3.9±1.1 | 4.0±1.2 | 3.9±1.1 | 0.787 |
| Creatinine (mg/dl) | 90.3±39.6 | 95.8±43.3 | 85.4±35.4 | 0.009 |
| eGFR | 70.5±23.5 | 66.2±22.4 | 74.4±23.9 | 0.001 |
| Blood urea | 7.6±6.4 | 7.9±4.5 | 7.2±7.8 | 0.336 |
| Potassium (mmol/L) | 4.0±0.6 | 4.0±0.7 | 4.0±0.5 | 0.922 |
| C-reactive protein | 3.3 (3.0-6.3) | 3.3 (3.0-7.5) | 3.3 (3.0-5.5) | 0.064 |
| **Medications data,** n (%) |  |  |  |  |
| Beta-blockers | 242 (60.5) | 112 (58.9) | 130 (61.9) | 0.546 |
| Diuretics | 192 (48.0) | 95 (50.0) | 97 (46.2) | 0.446 |
| MCRA | 129 (32.3) | 64 (33.7) | 65 (31.0) | 0.559 |
| Calcium channel blockers | 124 (31.0) | 53 (27.9) | 71 (33.8) | 0.201 |
| ACEI+ARB | 197 (49.3) | 90 (47.4) | 107 (51.0) | 0.474 |
| Statins | 335 (83.8) | 152 (80.0) | 183 (87.1) | 0.053 |
| **Echocardiography** |  |  |  |  |
| LAVI, mL/m2 | 42.6±6.2 | 43.6±5.8 | 41.8±6.3 | 0.003 |
| LVEDD (mm) | 47.4±5.6 | 47.7±5.3 | 47.0±5.9 | 0.246 |
| LVESD (mm) | 31.8±6.8 | 31.8±6.0 | 31.8±7.5 | 0.972 |
| LVEF (%) | 60.5±5.0 | 59.9±4.9 | 61.0±4.9 | 0.033 |
| e', cm/s | 7.0 (6.0-8.0) | 6.0 (5.8-8.0) | 7.0 (6.0-8.0) | 0.011 |
| Septal E/e’ | 15.0±3.0 | 15.4±2.9 | 14.7±3.1 | 0.020 |
| PASP (mmHg) | 38.7±11.4 | 39.9±12.5 | 37.6±10.4 | 0.044 |

BMI: body mass index; NYHA class: New York heart association; BP: blood pressure; CHD: coronary heart disease; HbA1c: glycated hemoglobin; ABG: admission blood glucose; SHR: stress hyperglycemia ratio; TSH: thyroid stimulating hormone; ALT: alanine aminotransferase; NT-proBNP: N-terminal pro–B-type natriuretic peptide; LDL: low-density lipoprotein; eGFR: estimated glomerular filtration rate; MCRA: mineralocorticoid receptor antagonist; ACEI+ARB angiotensin-converting enzyme inhibitors/angiotensin receptor blockers; LAVI: left atrial volume index; LVEDD: left ventricular end-diastolic diameter; LVESD: left ventricular end-systolic diameter; LVEF: left ventricular ejection fraction; e': peak LV velocity; E/e': mean septal velocity; PASP: pulmonary artery systolic pressure.

**Table S2.** Outcomes of study population according to SHR cut-off

|  | All patients  (n=400) | Patients with  SHR>0.99 (n=124) | Patients with  SHR≤0.99 (n=276) | P value |
| --- | --- | --- | --- | --- |
| Total Events | 190 (47.5) | 88 (71.0) | 102 (37.0) | <0.001 |
| All-cause mortality | 75 (18.8) | 35 (28.2) | 40 (14.5) | 0.001 |
| Cardiovascular mortality | 62 (15.5) | 28 (22.6) | 34 (12.3) | 0.009 |
| HF readmissions | 115 (28.8) | 53 (42.7) | 62 (22.5) | <0.001 |

SHR: stress hyperglycemia ratio; HF: heart failure.

**
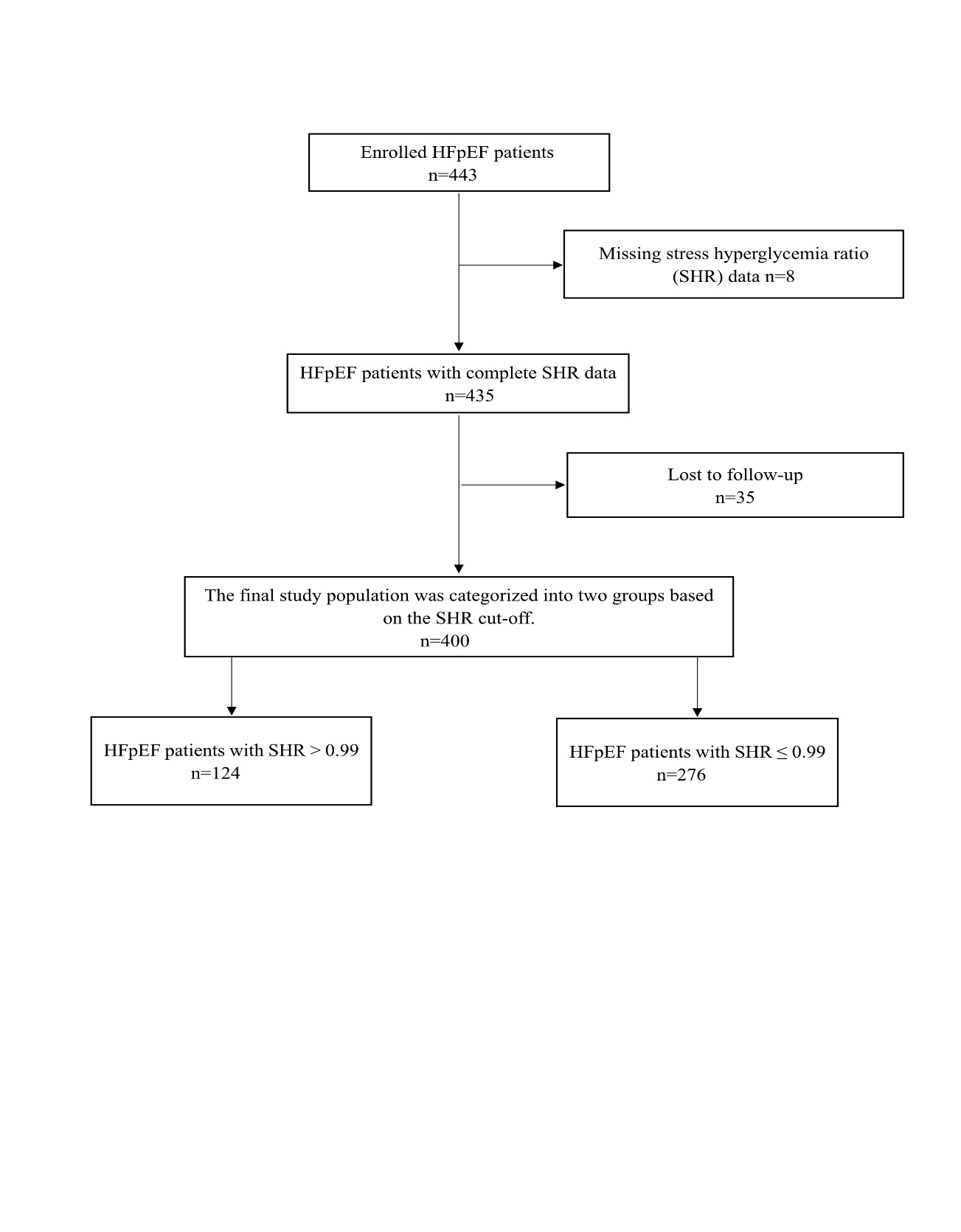
**

**Figure S1**: Flowchart of patients enrolled.

HFpEF: heart failure with preserved ejection fraction; SHR: stress hyperglycemia ratio.


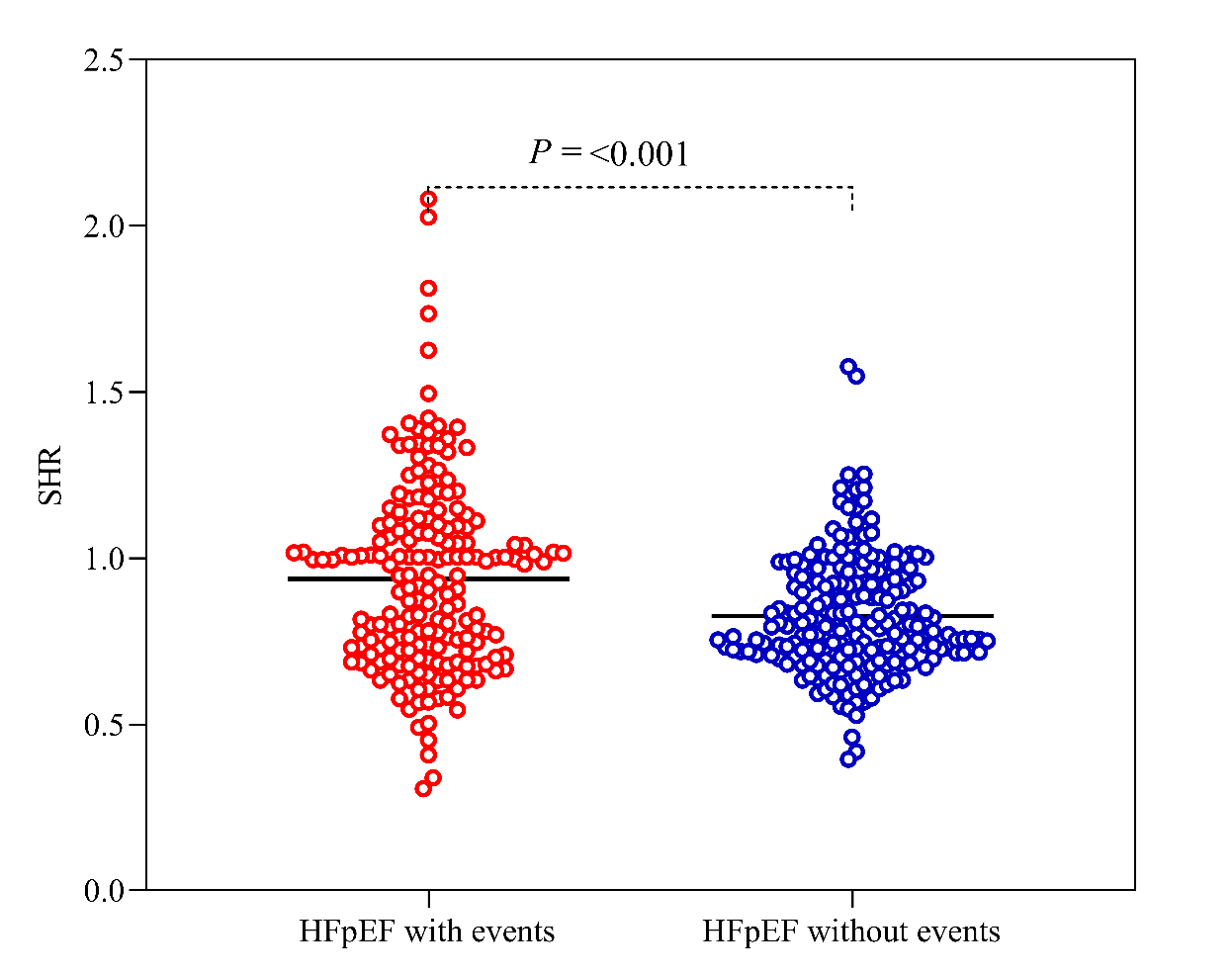


**Figure S2.** The distribution of SHR among HFpEF patients with and without events.

SHR: stress hyperglycemia ratio; HFpEF: heart failure with preserved ejection fraction


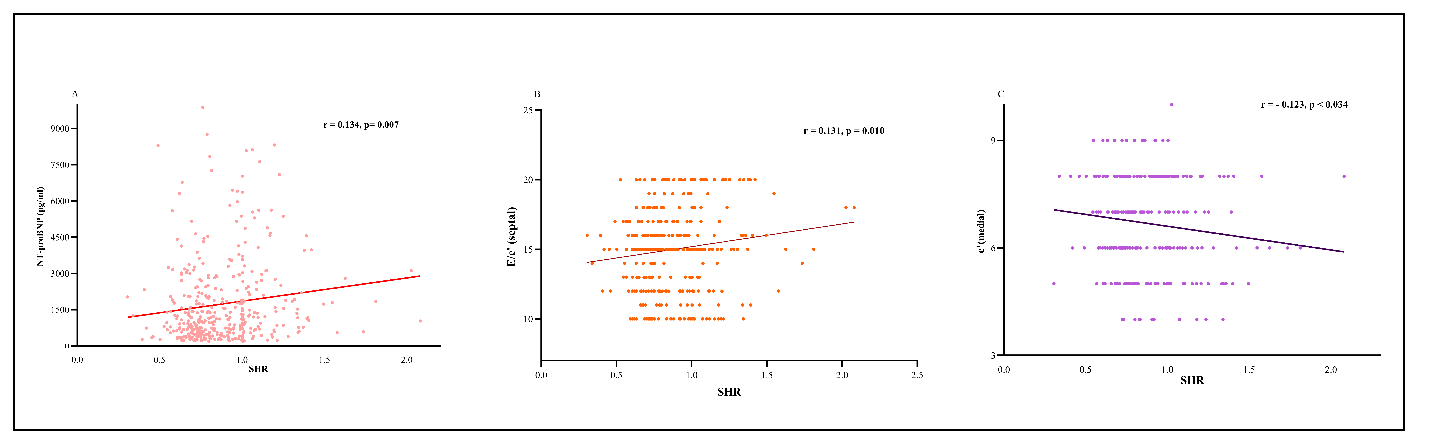


**Figure S3.** Relationship between SHR and HF parameters.

**A.** Correlation between SHR and NT-proBNP; **B.** Correlation between SHR and E/e'; **C.** Correlation between SHR and e'.

SHR: stress hyperglycemia ratio; NT-proBNP: N-terminal pro–B-type natriuretic peptide; E/e': mean septal velocity; e': peak LV velocity.


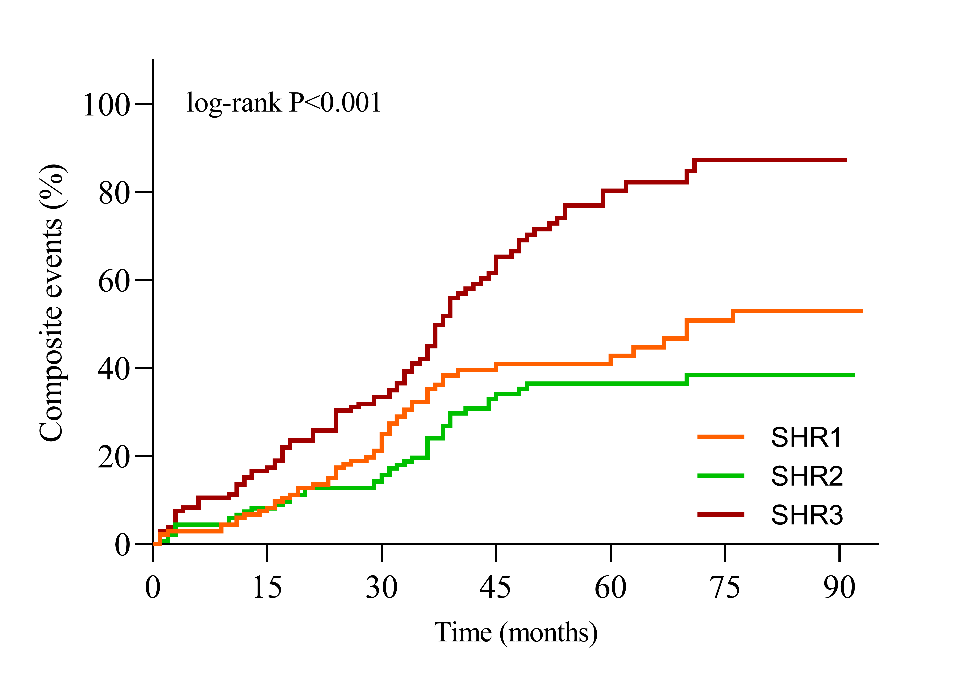


**Figure S4.** Outcomes stratified according to SHR tertiles.

SHR1: ≤0.74, SHR2: >0.74 - ≤0.98, SHR3: >0.98.

SHR: stress hyperglycemia ratio
